# A Rare Case of Bilateral Posterior Tibial Artery Aneurysm Presenting as Unilateral Acute Limb Ischemia

**DOI:** 10.1155/2019/5647380

**Published:** 2019-06-26

**Authors:** Andrew T. Hattam, Mayur Krishnaswamy

**Affiliations:** Vascular Surgery Unit, Department of Surgery, University Hospital Geelong, Barwon Health, Geelong, Victoria, Australia

## Abstract

True aneurysms of the tibial arteries are extremely rare. Of the few previously described tibial artery aneurysms, there are scant reports of isolated true aneurysms of the posterior tibial artery (PTA). In this report, we describe the second documented case of bilateral true PTA aneurysms. Unique aspects of this case are that the aneurysmal PTA were the only patent tibial arteries bilaterally, the aneurysms were degenerative in nature, and initial patient presentation was due to aneurysm thrombosis causing acute foot ischemia. The clinical and radiological features of this case, as well as surgical decision making and management, are discussed.

## 1. Introduction

Aneurysms of the tibial arteries are uncommon, with true aneurysms of the tibial arteries being extremely rare. Of the few previously described tibial artery aneurysms, there are scant reports of isolated true aneurysms of the posterior tibial artery (PTA) [1]. To our knowledge, there is only one report to date of a patient with bilateral true aneurysms of the PTA [2]. Herein, we describe a case of bilateral true PTA aneurysms presenting as unilateral acute foot ischemia. Informed consent has been granted by the patient to report this case.

## 2. Case Report

A 75-year-old female presented acutely with a history of sudden onset paraesthesia and ischemic rest pain involving the left foot and ankle. Motor function was retained, capillary and superficial venous return were reduced, the foot was cold, and there were no palpable pedal pulses. The calf remained soft to palpate. There was no history of palpitations or arrythmia, and the patient was in sinus cardiac rhythm both clinically and on electrocardiography. Plain chest radiography and computed tomography angiography (CTA) excluded a proximal arterial source of embolism. Furthermore, there was no previous trauma, (local or systemic) infection or inflammation, collagen, connective tissue or genetic syndromes, or arteritis; nor was there a family history of aneurysmal disease. Positive cardiovascular risk factors included hypercholesterolaemia and hypertension. Baseline haematological and biochemical analyses were normal, as were the renal and liver function. The creatinine kinase was within normal limits. The right foot was well perfused with a palpable PTA pulse.

**Figure 1:**
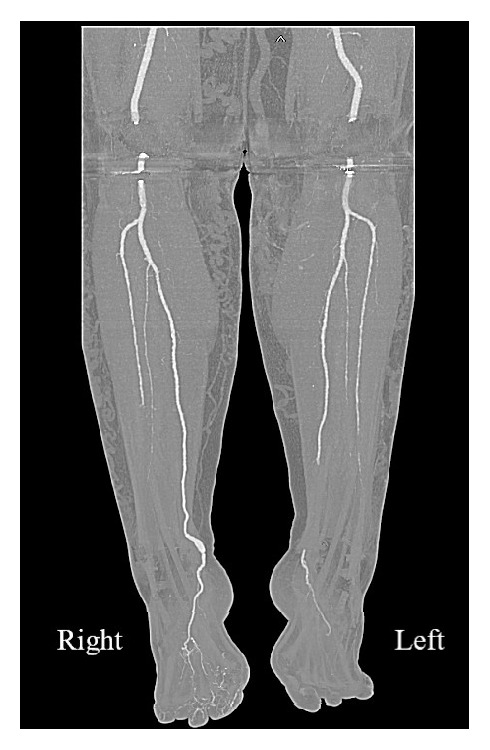
Preoperative reconstructed computed tomography angiography of the tibial arteries bilaterally, demonstrating abrupt occlusion of the left posterior tibial artery and a right-sided posterior tibial artery aneurysm. Please note that artefact from bilateral knee prostheses obstructs visualising normal flow within the popliteal arteries in this image.

CTA demonstrated no significant stenosis in the supragenicular vessels bilaterally. On the symptomatic left side, the tibioperoneal trunk (TPT) and anterior tibial artery (ATA) were patent proximally, with the ATA and peroneal artery occluding in the distal calf. The dorsalis pedis was occluded; however, the plantar arteries reconstituted. Dominant flow to the foot was via the PTA, which occluded in the distal calf (Figure 1). These findings were considered reflective of embolic disease within the left-sided tibial arteries. The asymptomatic right leg demonstrated dominant flow to the ankle via the PTA. The ATA and peroneal arteries occluded in the midcalf.

**Figure 2:**
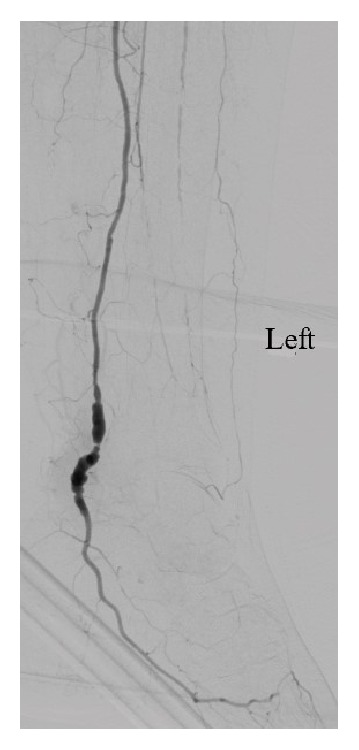
Angiography of the left posterior tibial artery aneurysm after thrombectomy.

**Figure 3:**
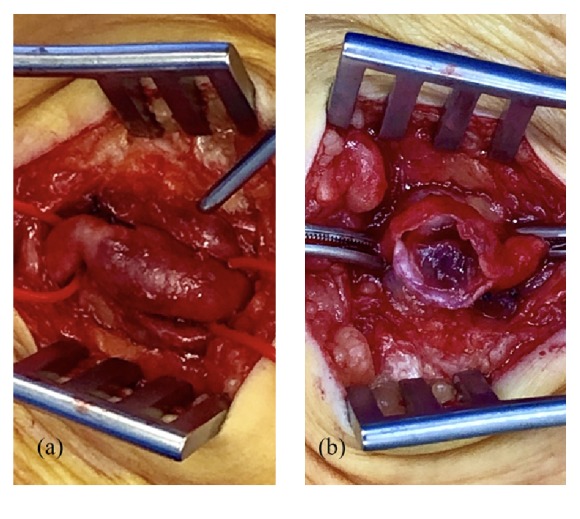
Intraoperative images demonstrating exposure of the posterior tibial artery aneurysm (a) and containing intramural thrombus (b).

**Figure 4:**
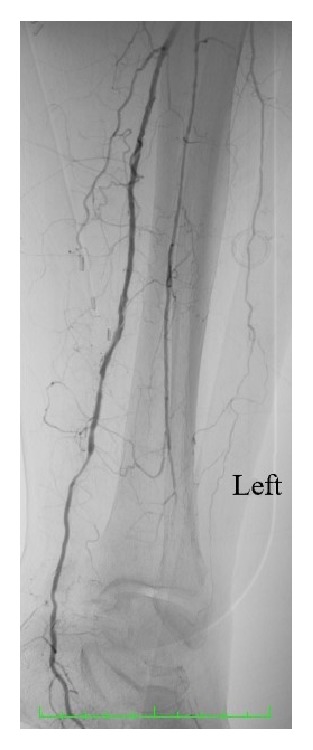
Postoperative angiography demonstrating flow to the left foot through the interposition long saphenous vein graft.

Due to the acute nature of the patient's symptoms, emergent thromboembolectomy of the popliteal and tibial arteries was performed via a medial approach to the distal popliteal artery. Selective thromboembolectomy using Fogarty catheters for each tibial artery was unsuccessful. Urokinase was also injected into each tibial artery without effect. Subsequent exposure of the dominant PTA at the ankle demonstrated a thrombosed 10mm diameter aneurysm. A PTA thrombectomy was performed and subsequent angiography demonstrated flow to the foot via the aneurysmal PTA (Figure 2). The aneurysm was opened and demonstrated a mixture of old, organised, and fresh thrombus (Figure 3). The PTA aneurysm was excised and thrombectomy established adequate inflow and backflow to the excised PTA segment. The PTA was repaired using an interposition long saphenous vein graft (Figure 4). Postoperatively, the PTA pulse was present, the foot had brisk capillary refill, and motor and sensory function normalised. Follow-up at 16 months demonstrated the patient to have a well perfused left foot with a palpable PTA pulse.

Although the patient's contralateral foot was asymptomatic, she opted to have this repaired electively considering the aneurysmal right PTA aneurysm was the single arterial supply to the foot. Repair was performed via excision of the 8mm PTA aneurysm and a spatulated primary end-to-end PTA anastomosis. Completion angiography was satisfactory, and the right foot was well perfused with a palpable PTA pulse postoperatively. Histopathology of the right-sided PTA aneurysm demonstrated findings consistent with atherosclerotic degenerative aneurysmal disease. Specifically, there was intimal thickening, extensive lamina disruption, and broad areas of media replaced by collagenous fibrous tissue, with calcification of the media. There was no inflammation or mural necrosis seen.

## 3. Discussion

Most tibial aneurysms present as posttraumatic false aneurysms [1, 3, 4]. Other described aetiological mechanisms include autoimmune, infection, collagen and/or connective tissue disorders, genetic syndromes, and arteritis [5].

The number of isolated tibial aneurysms in the English literature is estimated at <50 cases, with <15 cases of isolated PTA aneurysms [1, 3]. Two previous reports have described bilateral PTA aneurysms. However, Tressider et al. [6] described mycotic aneurysms of the TPT, which involved the proximal PTA and peroneal arteries. Katz et al. [2] described bilateral PTA aneurysms with progressive bilateral calf swelling and pain. Immunohistological examination of tissue from this patient demonstrated the aneurysms to be due to a nonspecified immune-complex disease and associated collagen disorder within the arterial walls. Histology of the case described here demonstrated simple degenerative aneurysm disease. Assuming the pathological processes was mirrored in the contralateral aneurysm, this case would be the first case of bilateral degenerative PTA aneurysms in the English literature.

Most tibial aneurysms present as asymptomatic medial ankle swellings [4]. Due to their rarity, the natural history of tibial artery aneurysm disease remains incompletely characterised. Previous reports suggest symptomatic presentations are more commonly due to aneurysm thromboembolism (as occurring in this case) rather than rupture [5]. In assessing the patient for tibial artery arterial aneurysms, ultrasound Doppler studies [1], formal angiography [3], and magnetic resonance angiography [5] have been described. However, as demonstrated on CTA (Figure 1) in this case, it is difficult to characterise small vessel, noncalcific, distal tibial artery aneurysms using such imaging modalities once acute thromboembolism has occurred. Moreover, as in this case, concomitant occlusive disease of the other tibial arteries may mimic embolic disease, making diagnosis of such rare pathology more difficult. Furthermore, the very low incidence of bilateral aneurysm disease is unlikely to be able to guide clinical suspicion of such cases.

Given the rarity of tibial artery aneurysms, a standard treatment approach has yet to be established [4]. Previous authors have described relatively long-term surveillance of tibial artery aneurysms without adverse sequelae [7, 8], while others suggest surgical repair irrespective of symptoms [4]. In the emergency setting, ligation of the aneurysmal artery has been advocated [4]. However, as at least one uncompromised tibial artery is required to adequately perfuse the foot [9], the presence of ATA and peroneal artery occlusions in this case necessitated emergent reconstruction of the aneurysmal PTA to salvage the acutely ischemic foot.

The asymptomatic right leg PTA aneurysm was subsequently managed with elective surgical repair. This was performed on the basis of (1) the established natural history of the left-sided PTA aneurysm, (2) the PTA being the sole arterial supply to the foot, and (3) the established thromboembolic risk of intramural thrombus within tibial artery aneurysms identified radiologically [10].

In conclusion, tibial artery aneurysms are extremely rare and their aetiology is widely variable. Diagnosis in the emergency setting is difficult, both clinically and radiologically, after acute thromboembolic evolution. The case described herein is the second case of bilateral isolated PTA aneurysms and the first of degenerative aetiology presenting with acute foot ischemia. Consensus on the management of tibial artery aneurysms has yet to be defined. Though ligation of the aneurysmal artery is a valid surgical option in the emergency setting, we suggest reconstruction of the aneurysmal artery in circumstances where it is the sole arterial supply to the foot. The most difficult aspect of this case was diagnosis of an extremely rare tibial artery aneurysm in the acute setting. Although rare, clinicians should be mindful of the potential for tibial aneurysms at routine clinical examination, as diagnosis and management appear to be easier and safer, in the elective setting.
